# Peste des Petits Ruminants Virus, Eastern Asia

**DOI:** 10.3201/eid2012.140907

**Published:** 2014-12

**Authors:** Ashley C. Banyard, Zhiliang Wang, Satya Parida

**Affiliations:** Animal and Plant Health Agency, Weybridge, UK (A.C. Banyard);; Ministry of Agriculture of the People’s Republic of China, Qingdao, China (Z. Wang);; The Pirbright Institute, Pirbright, UK (S. Parida)

**Keywords:** peste des petits ruminants virus, emergence, morbilliviruses, viruses, China, Asia

**To the Editor:** Peste des petits ruminants virus (PPRV) is reported globally with increasing frequency. Recently, PPRV has been detected in areas where it is considered endemic and in neighboring areas where it previously has not been reported. The reporting of “first cases” in regions where PPRV has been considered endemic is of little surprise and perhaps represents increased interest both in agricultural practices and diagnostic capacity (*1*–*3*). Increased development of the small ruminant health sector, expanding small ruminant populations, increased trade movement, and rinderpest eradication might all have affected PPRV detection (*4*). The latter theory is of great interest because rinderpest eradication may have affected the epidemiology of PPRV through complete removal cross-protective rinderpest infection of small ruminants and cessation of small ruminant vaccination with the rinderpest vaccine to prevent PPRV infection. Indeed, the potential effect of rinderpest eradication on PPRV epidemiology should not be understated because it might have profoundly affected PPRV emergence by enabling free transmission and spread of the virus, perhaps overcoming the genetic and geographic bottlenecks created by rinderpest circulation and/or the use of rinderpest vaccines. In addition, rinderpest eradication has highlighted the possibility that PPRV could be eradicated by using comparable systems and tools (*5*).

Historically, PPRV has been identified across much of the developing world; genetic analyses has grouped viruses into 4 lineages that were originally thought to be phylogeographically restricted (*6*). However, in recent years, lineages of PPRV have apparently emerged in new areas. This has been most convincingly demonstrated with the detection of lineage IV virus—a lineage thought restricted to the Indian subcontinent and the Middle East—across northern and central Africa (Technical Appendix Figure) (*7*,*8*). However, reporting of PPRV in areas where it has not been previously detected is perhaps of greater interest. This is increasingly the case across southern and eastern Asia where virologic and serologic evidence of circulating PPRV has been reported (*6*)

During 2014, PPRV caused extensive agricultural losses across China. Although regions within China had previously reported relatively small outbreaks, during December 2013–June 2014, the virus appears to have greatly extended its distribution. In 2007, PPRV was detected for the first time in the Ngari region of southwestern Tibet (*9*). This emergence was thought to have arisen through the circulation of mild forms of PPRV infection and the unfamiliarity of agricultural workers and professionals (e.g., veterinarians, farmers, livestock owners) with the disease and the inability to differentiate between mild forms of PPRV infections and other diseases of small ruminants. PPRV returned in 2008 and 2010 and was controlled by using stamping-out procedures, animal movement control, and increased screening of herds. The disease was controlled without the use of vaccines in 2008; vaccination was used in 2010 (*10*).

Three years passed without reports of PPRV infections in Tibet or elsewhere in China before the virus was detected in Xinjiang, China’s largest administrative division, in December 2013. Xinjiang, an area of 1.6 million km^2^, borders Afghanistan, India, Kazakhstan, Kyrgyzstan, Mongolia, Pakistan, Russia, and Tajikistan, several of which have reported PPRV infection. Within 2 months, PPRV had caused 3 outbreaks with rates of illness (and death) of 17% (2%), 58% (11%), and 79% (19%), respectively. Measures to contain these outbreaks were implemented as in 2007; however, during April and May 2014, the number of PPRV outbreaks increased sharply across much of China, including in Anhui, Guizhou, Guangxi, Hubei, Hunan, Shanxi, Xinjiang, Yunnan, and Zhejiang Provinces (Figure). The origin of these outbreaks remains undefined; however, the ability of the virus to circulate causing mild clinical disease and its presence in numerous bordering countries suggest several possibilities regarding the source of disease, including spread from the original China outbreaks. Similarly the threat of further spread from China to neighboring countries cannot be ignored.

**Figure:**
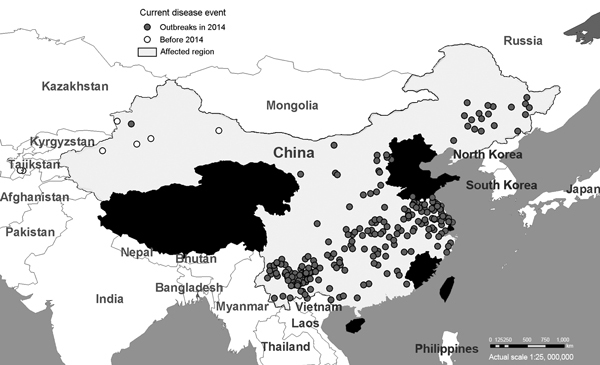
Outbreaks of peste des petits ruminants virus (PPRV) across China during December 2013–May 2014. Data are from ProMed alerts during the period described (*10*).

Once the current situation has been resolved, full genetic analysis of the viruses causing the outbreaks should be conducted because it might indicate the direction of spread. A further area of interest is the application and choice of control measures. Although predicting the spread of a viral pathogen is impossible, especially across the vast distances involved in the current reports, the experiences in China might influence future responses to incursions of PPRV into areas where PPRV previously has not been documented. The current lack of disease in areas where vaccination was reported in 2010 could explain the continued absence of disease from such areas while other regions are significantly affected (*10*). Effective vaccines against PPRV have been available for decades and will now, as both reactive and preventive tools, aid in controlling and preventing onward transmission of this viral pathogen. Once the situation in China is under control, where this emerging infection of small ruminants will appear next remains to be seen.

Technical Appendix Maps showing the historic and current detection of peste des petits ruminants virus lineage IV across Africa.
