# Supplementary material for: Peste des Petits Ruminants Virus, Eastern Asia
Source: Emerg Infect Dis. 2014 Dec;20(12):2176–8. doi: 10.3201/eid2012.140907 (PMC4257806; doi:10.3201/eid2012.140907)
Supplement: Technical Appendix — Maps showing the historic and current detection of peste des petits ruminants virus lineage IV across Africa. [file 14-0907-Techapp-s1.pdf]

Article DOI: http://dx.doi.org/10.3201/eid2012.140907

Peste des Petits Ruminants Virus, Eastern Asia

Technical Appendix

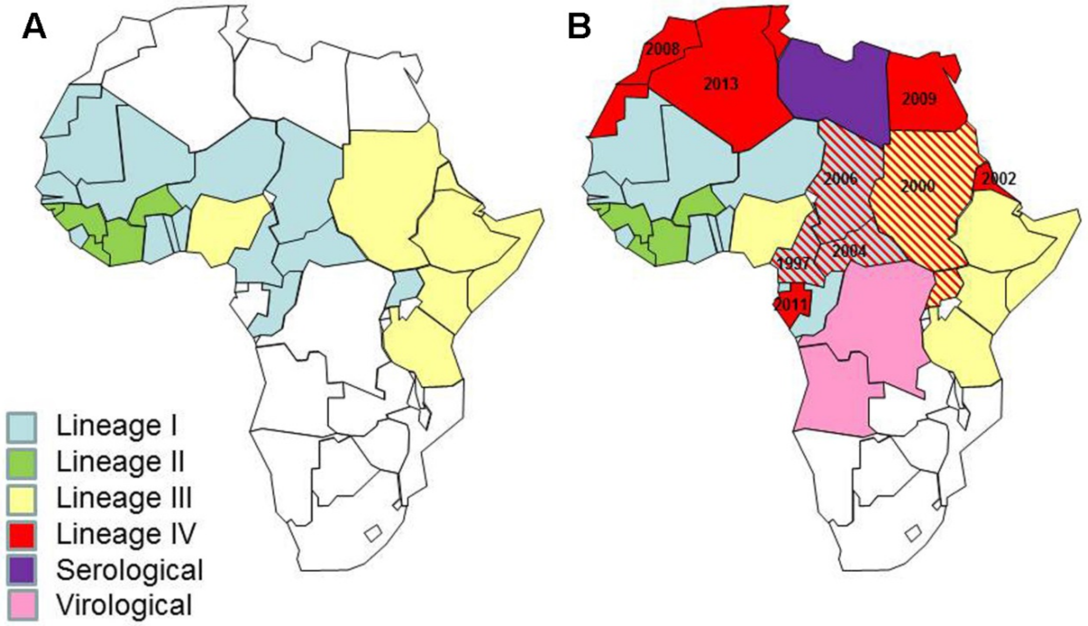

Technical Appendix. Maps showing the historic and current detection of peste des petits ruminants virus (PPRV) lineage IV across Africa. A) Lineages detected across Africa before the detection of lineage IV in Africa in 1997. B) Emergence of lineage IV across Africa during 1997–2014. Lineages are numbered as detailed in (*1*). Dates of first report of lineage IV are shown in B. Hatched shading indicates regions where multiple lineages may be circulating (data from *1*–*3*).

Reference

1. Banyard AC, Parida S, Batten C, Oura C, Kwiatek O, Libeau G. Global distribution of peste des petits ruminants virus and prospects for improved diagnosis and control. J Gen Virol. 2010;91:2885–97. PubMed http://dx.doi.org/10.1099/vir.0.025841-0

2. Kwiatek O, Ali YH, Saeed IK, Khalafalla AI, Mohamed OI, Obeida AA, et al. Asian lineage of peste des petits ruminants virus, Africa. Emerg Infect Dis. 2011;17:1223–31. PubMed http://dx.doi.org/10.3201/eid1707.101216

3. ProMedMail. Peste des petits ruminants—China (11): Sheep, goat, spread, OIE, request for information. ProMed 2014 May 5. http://www.promedmail.org, archive no. 20140511.2465094.
